# Information Technology–Assisted Treatment Planning and Performance Assessment for Severe Thalassemia Care in Low- and Middle-Income Countries: Observational Study

**DOI:** 10.2196/medinform.9291

**Published:** 2019-01-23

**Authors:** Rajat Kumar Agarwal, Amit Sedai, Kumari Ankita, Lalith Parmar, Rakesh Dhanya, Sunil Dhimal, Reshma Sriniwas, Ashwini Gowda, Pooja Gujjal, Pushpa H, Suman Jain, J Dasaratha Ramaiah, Sujata Jali, Neelavva Rayappa Tallur, Stalin Ramprakash, Lawrence Faulkner

**Affiliations:** 1 Jagriti InnoHealth Platforms Private Ltd Bangalore India; 2 Sankalp India Foundation Bangalore India; 3 Project Samraksha Rashtrotthana Parishat Bangalore India; 4 Indira Gandhi Institute of Child Health Bangalore India; 5 Thalassemia and Sickle Cell Society Hyderabad India; 6 Rural Development Trust Hospital Ananthpur India; 7 Jawaharlal Nehru Medical College Belgaum India; 8 Sankalp-People Tree Centre for Pediatric Bone Marrow Transplant People Tree Hospitals Bangalore India; 9 Cure2Children Foundation Florence Italy

**Keywords:** β-thalassemia major, health information technologies, health planning, patient outcome assessment

## Abstract

**Background:**

Successful models of information and communication technology (ICT) applied to cost-effective delivery of quality care in low- and middle-income countries (LMIC) are an increasing necessity. Severe thalassemia is one of the most common life-threatening noncommunicable diseases of children globally.

**Objective:**

The aim was to study the impact of ICT on quality of care for severe thalassemia patients in LMIC.

**Methods:**

A total of 1110 patients with severe thalassemia from five centers in India were followed over a 1-year period. The impact of consistent use of a Web-based platform designed to assist comprehensive management of severe thalassemia (ThalCare) on key indicators of quality of care such as minimum (pretransfusion) hemoglobin, serum ferritin, liver size, and spleen size were assessed.

**Results:**

Overall improvements in initial hemoglobin, ferritin, and liver and spleen size were significant (*P*<.001 for each). For four centers, the improvement in mean pretransfusion hemoglobin level was statistically significant (*P*<.001). Four of five centers achieved reduction in mean ferritin levels, with two displaying a significant drop in ferritin (*P*=.004 and *P*<.001). One of the five centers did not record liver and spleen size on palpation, but of the remaining four centers, two witnessed a large drop in liver and spleen size (*P*<.01), one witnessed moderate drop (*P*=.05 for liver; *P*=.03 for spleen size), while the fourth witnessed a moderate increase in liver size (*P*=.08) and insignificant change in spleen size (*P*=.12).

**Conclusions:**

Implementation of computer-assisted treatment planning and performance assessment consistently and positively impacted indexes reflecting effective delivery of care to patients suffering from severe thalassemia in LMIC.

## Introduction

Internet access has grown dramatically in low- and middle- income countries (LMIC) [1]; however, whether improved access to information and communication technologies (ICTs) can improve outcomes is an ongoing debate [2-6]. In fact, the application of information systems to improve access to health care in developing countries has faced many failures related to factors such as mismatch between ICT designs and local user actual needs, system maintenance, upgrades, and repairs [7-9]. The shifting need in many LMIC from infectious diseases and primary health care to chronic noncommunicable diseases (NCD) requiring tertiary care and specialized medical competencies underscores the urgent need to improve access to qualified consultation, patient monitoring, and outcomes assessment. Context-appropriate, friendly, and user-driven ICT systems may play a key role in achieving those goals.

Health care continues to be a challenge in India with only 7 physicians and 17.1 nurses for every 10,000 people against a global mean of 17.1 and 25.3, respectively [10]. Overburdened and underfunded health care prioritizes situations requiring acute medical care. The emergence of NCD in LMIC is driving many families into poverty (an estimated 40 million people a year only in India [11,12]), and the issue of consistency and value assessment in medical care becomes increasingly critical [13]. Last but not least is the need of data management to strengthen health care systems, support governments and other health-providing bodies, and improve research and higher medical education [14,15].

In the pediatric age group, severe hemoglobinopathies, namely severe thalassemia and sickle cell disease (SCD), represent the most frequent life-threatening NCD globally and are a major burden to affected families and health care systems [16-21]. It has been estimated that if the survival rate of children with SCD in Africa increases to only 50% from the current African norm, more than 6 million Africans will be living with SCD [22]. In India, severe thalassemia—a thalassemia syndrome with an inability to spontaneously maintain hemoglobin levels at or above 7 g/dL—is still the largest challenge in the spectrum of hereditary disorders [23-25].

Significant improvements have been made in the care and management of severe thalassemia in the last few decades [26,27]. However, the World Health Organization estimated that only about 9.6% of transfusion-dependent patients suffering from hemoglobin disorders are actually transfused in Southeast Asia [28] and management decisions are often driven by immediate short-term needs rather than established best practices and guidelines. The cumulative impact of years of improper management results in an avoidable morbidity and mortality burden [24,29,30].

The primary objective of care and management of severe thalassemia is to maintain adequate hemoglobin levels while controlling iron overload [31]. In the context of the developing world, it also includes the need to reverse the impact of ineffective and insufficient management from the past [32,33]. Pretransfusion hemoglobin level is the marker for adequacy of blood transfusions. Serum ferritin is a widely used and acceptable marker for iron overload [34,31]. Liver and/or spleen enlargement also reflect inadequate management and are quite easy to monitor by clinical examination; in fact, liver size on palpation remains one of the most relevant predictors of bone marrow transplant outcome [35,36].

With over 10,000 new patients with severe thalassemia estimated to be born in India alone every year [37], the challenge is to create smart delivery systems that reduce the burden on health care professionals while improving outcomes. We used information technology as an enabler to assist delivery of care to severe thalassemia patients.

## Methods

### Study Design

We measured the impact of computer-assisted treatment planning and performance assessment in five centers in India that adopted ThalCare between 2011 and 2017, namely Indira Gandhi Institute of Child Health in Bangalore, Project Samraksha (Rashtrotthana Parishat) in Bangalore, Thalassemia and Sickle Cell Society in Hyderabad, Rural Development Trust in Ananthpur, and Jai Shivshakti Center for Thalassemia (Jawaharlal Nehru Medical College) in Belgaum.

We selected four quality-of-care indicators: (1) pretransfusion hemoglobin level, (2) serial serum ferritins, (3) liver size, and (4) spleen size. First and fifth quarterly means of each parameter where compared with the intent of quantifying the impact made after a year of use. A total of 4709 visits, 3782 liver measurements, 3825 spleen measurements, and 957 ferritin tests were included in the study. The setup of the centers, staffing, patient age, and gender are summarized in Table 1.

### Software Platform Description

The Web-based health ICT platform ThalCare is designed specifically to cater to data management and analytics needs of centers involved in care of severe thalassemia. It was built using free and open-source tools including LAMP (Linux, Apache, MYSQL, and PHP) and software stack using Drupal. This cloud-hosted app is accessible through any internet-enabled device with user-specific password-protected accounts. The system was secured and maintained in line with best practices for Web-based health care software. The app was remotely backed up periodically, the software stack was kept updated and guarded against known vulnerabilities, all user activity was logged, and inactivity-triggered timeouts enforced. The users had role-wise filtered access to data related to their own centers.

**Table 1:** Details of the centers^a^ and enrollment information.

Category		IGICH	SAM	TSCS	RDT	JSCT
Host institution		Attached to a major academic children’s hospital	Attached to a stand-alone blood bank	Attached to a stand-alone blood bank	Attached to a rural hospital	Attached to a medical college
Setup		Government	Private (nonprofit)	Private (nonprofit)	Private (nonprofit)	Private (nonprofit)
Began using ThalCare		Nov 2011	Aug 2013	Nov 2014	Aug 2015	Mar 2016
Doctors, n		1 part-time	1	2	1 part-time	1 part-time
Nurses, n		1	1	2	1	2
Coordinators, n		1	1	2	0	0
Patients, n^b^		154	144	668	88	185
Patient age (years), median^b^		6.5	9.1	6.6	8.4	8.4
**Patient sex, n^b^**						
	Male	96	68	58	399	117
	Female	58	76	30	269	68

^a^IGICH: Indira Gandhi Institute of Child Health; JSCT: Jai Shivshakti Center for Thalassemia; RDT: Rural Development Trust; SAM: Project Samraksha; TSCS: Thalassemia and Sickle Cell Society.

^b^Data taken at the end of the fifth quarter of adoption of ThalCare.

The system allows capturing patient’s contact details, demographic details, socioeconomic information, past severe thalassemia management history, vaccination status, related medical details of other members of the family, etc, at enrollment. Thereafter every patient intervention, including vital signs recordings, clinical notes, blood transfusions, medical prescriptions, laboratory investigations, complications and their management, and growth-related information, are captured on the ICT platform. With comprehensive use of technology, the units are essentially paperless in their operations.

The majority of nurses and coordinators who entered most of the data had never used computer systems before and started using the system after one day of training. In the initial stages, data were reviewed daily and feedback was provided by phone or online sessions. A technology support team was always available on call and provided timely assistance. Within the first month of using the system, all relevant medical data were available on the system.

### Scheduling

Requests are sent to the blood bank specifying when blood will be needed, and visits are scheduled sufficiently in advance to match blood availability thus minimizing idle time for patients. An alert is generated for a missed appointment prompting the staff to contact families and reschedule visits.

### Treatment Planning

When a patient comes for review, the caregiver updates the clinical record and reviews the system records for alerts on required interventions. The staff plan the day based on the inputs from patient indicators (Textbox 1). The system calculates the volume of blood to be transfused for each child based on preprogrammed configurable formulas; it enables investigations to be done at specified intervals thus facilitating early detection of complications, generates laboratory investigation forms and labels, and interfaces with multiple external laboratory information systems to place requests and retrieve reports.

**Indicators available on the information and communication technology platform.**

**Biochemistry**: displays parameters outside reference range and reminds of the need to repeat the test

**Bone marrow transplant eligibility**: checks if one or more healthy sibling is available and if age is less than prescribed cut-off age

**Complete blood count**: warns of cytopenia

**Chelation**: notifies if chelation information is not available and indicates if there is a need to start chelation; also suggests if dose of the chelator is outside the prescribed ranges

**Ferritin**: alerts on the grading of the ferritin levels and give reminders for tests

**Hemoglobin**: alerts if the patient has been discharged with the inappropriate hemoglobin levels in the past visits

**Serology**: alerts if the patient has one or more transfusion-transmitted infections and reminds for repeating the tests

**Transfusion**: alerts on the rate of fall of hemoglobin per week and warns of ineffective blood transfusion therapy

**Ultrasound**: alerts on hepatosplenomegaly as per grading and reminds to repeat the tests

**Vaccination**: alerts on vaccination status

### Care Management

When meeting a patient, the caregiver has a summary of alerts and the system suggests possible interventions. Centers can deactivate certain alerts and only those which require action are visible to avoid alert fatigue [2,38]. Categorized clinical notes allows doctors to find context-appropriate details. Continuous dose adjustment is critical to the success of iron chelation therapy [39,40]: the system prompts if the medication doses do not agree with treatment protocols based on laboratory results and patient’s weight. The system also tracks compliance to treatment by tracking the purchase/issue of drugs [41] dispensed directly by centers.

Change in patient’s growth, blood counts, and other laboratory values are tracked over time. The system automatically converts height, weight [42,43], and ultrasound measures [44] into age-adjusted *z* scores and presents them as charts for better visualization. Laboratory values are color-coded using the red-amber-green methodology, and tooltips provide reference ranges. Subsequent patient visits are also scheduled based on the volume of blood transfused and the estimated posttransfusion hemoglobin levels.

All complications are recorded and tracked on the system enabling a comprehensive understanding of patient-specific medical needs.

### Networking and Collaboration

The ICT platform enables point-of-care professionals to seek advice from more experienced centers. Queries posted on the system are answered asynchronously by experts who are part of the network and have access to the entire clinical history. This enables relatively junior local doctors to team up with specialists and enhance clinical management. Periodic review of the center’s progress and challenges is also done through online meetings.

### Blood Bank Coordination

The ICT platform allows the clinical team to monitor qualitative and quantitative aspects of associated blood bank support including time elapsed between collection and issue of blood, leukoreduction/depletion, blood request processing time, unavailability of blood locally, demand for replacement blood, and transfusion reaction.

### Outcomes and Performance Monitoring

In addition to severe thalassemia treatment planning and daily workflow organization, the ICT platform can generate outcome analysis and drive quality control. The Thal Report Card is a periodic autogenerated ICT report which summarizes the status of the patients at the center and allows for a quick review of the center’s performance. The report is automatically emailed to the health care providers and administrators every week and at the beginning of the month so that everyone involved has a clear view of the status and challenges.

Adverse changes in metrics in the report card act as triggers for quality improvement at the center. Objective evaluation allows discussions to be based on quantified hard data. Most centers have weekly meetings to discuss unit-specific issues and monthly meetings to discuss overall direction and progress. Transparent reporting is also a motivator for involved personnel, who get immediate visibility and recognition, and has allowed nursing staff at these institutions to take on a more active role in patient care and monitoring. The centers also participate in annual preview with peer benchmarking and sharing of best practices.

### Transparency, Accountability, and Community Engagement

Another key aspect of the Thal Report Card is that it enables data-driven communication with funding agencies, regulators, administrators, and patient groups to monitor progress. Positive changes act as a catalyst to further aid resource mobilization, while transparent reporting builds accountability leading to continuous quality improvement.

### Patient App

**Figure 1:**
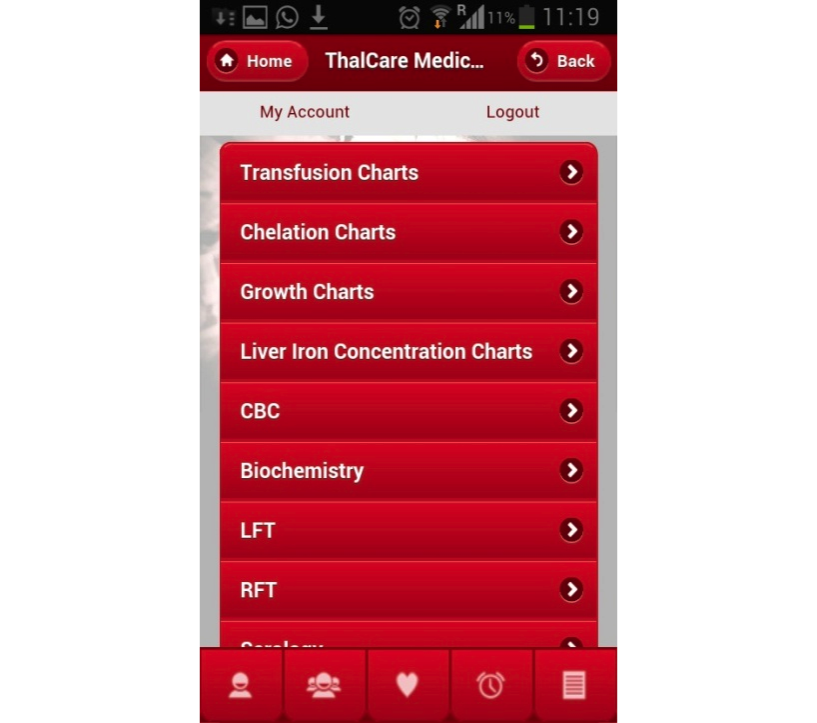
Screenshot of the patient app.

The ICT platform allows patients to log in and track their medical progress, see laboratory reports and appointments, and interact with the treating team leading to enhanced awareness and involvement (Figure 1). This helps maintain accurate and updated personal medical information while eliminating the need for paper documents and vastly simplifying record keeping. However, this was useful only for the more educated patients/families.

### Statistical Analysis

Data collected from the ICT platform was summarized using MS Excel. The mean values of the pretransfusion hemoglobin levels and liver and spleen sizes were compared using Welch *t* test (independent two-sample, two-tailed assuming unequal size and unequal variance) and ferritin was compared using matched-pair *t* tests using R version 3.3.2.

## Results

### Adequacy of Blood Transfusions

Overall improvement in pretransfusion hemoglobin was highly significant (*P*<.001) and the same was seen in four centers (Table 2). The improvement was statistically significant in four (*P*<.001), whereas the fifth center saw a marginal drop of 0.1 gm/dL (*P*=.09).

The number of visits increased from a mean of 0.7 (range 0.5-1.5) visits per month to 1.1 (range 0.8-1.4) visits per month. Four centers sourced all blood from the attached blood banks. The need for patients to get blood from outside fell from 17% to 11% in the fifth center. The mean duration between collection and issue of blood was 4.3 (SD 2.9) days. In three centers, blood was processed within 3 hours; one center did not measure this time and the remaining center took 4.7 hours. Transfusion- transmitted infection prevalence was 1.8% (range 0%-2.9%). Of this, 1.7% was detected at registration with ThalCare and 0.12% was detected subsequently.

### Iron Overload Management

The overall reduction in mean ferritin levels was significant (*P*<.001). Two centers achieved significant reduction in mean ferritin levels (*P*<.001 and *P*=.004), two had an insignificant drop (*P*=.80 and *P*=.50), and the remaining one had an insignificant increase (*P*=.80) (see Table 2).

Of the 983 changes made to chelation drug doses, 521 (53.0%) were made as a response to system-generated alerts for dose change.

### Response to Liver and Spleen Size on Palpation

Overall, there was highly significant improvement in liver (*P*<.001) and spleen (*P*<.001) sizes. One center did not record liver and spleen size. Two witnessed highly significant drops in liver and spleen size (*P*<.01), one witnessed moderate drop (*P*=.05 for liver; *P*=.03 for spleen), while the fourth witnessed increase in liver size (*P*=.08) and insignificant change in spleen size (*P*=.12) (see Table 2).

**Table 2:** Change in the key parameters in the first year of adoption of ThalCare.

Group		Pretransfusion Hb (g/dL)			Ferritin (ng/mL)			Liver size (cm)			Spleen size (cm)		
		Mean (SD)	n	*P* value	Mean (SD)	n	*P* value	Mean (SD)	n	*P* value	Mean (SD)	n	*P* value
**Quarter 1**													
	Overall	8.0 (1.6)	1009	—^a^	4940 (4628)	427	—	2.4 (2.4)	869	—	3.5 (3.8)	863	—
	IGICH^b^	7.1 (1.4)	151	—	3856 (3050)	95	—	3.1 (2.6)	154	—	3.1 (3.3)	154	—
	SAM^c^	8.2 (2.0)	232	—	5376 (2843)	99	—	3.8 (2.4)	161	—	5.6 (5.4)	161	—
	TSCS^d^	8.2 (1.3)	405	—	3281 (1537)	42	—	1.2 (1.3)	364	—	2.4 (2.8)	361	—
	RDT^e^	7.9 (1.6)	221	—	3959 (4051)	29	—	2.9 (2.7)	190	—	4.1 (3.7)	187	—
	JSCT^f^	7.5 (1.7)	369	—	5916 (6380)	162	—	—	—	—	—	—	—
**Quarter 5**													
	Overall	8.3 (1.3)	2891	<.001	3517 (2262)	530	<.001	1.6 (2.0)	2802	<.001	2.2 (3.0)	2798	<.001
	IGICH	7.7 (1.4)	214	<.001	2849 (1647)	151	.004	0.9 (1.9)	245	<.001	0.8 (1.9)	245	<.001
	SAM	8.7 (1.3)	374	<.001	4569 (2916)	130	.80	3.2 (1.9)	345	.01	2.3 (3.5)	339	<.001
	TSCS	8.1 (1.3)	1985	.09	3363 (2039)	88	.80	1.3 (1.7)	1886	.08	2.2 (2.9)	1891	.12
	RDT	9.1 (1.3)	318	<.001	3404 (2328)	54	.50	2.4 (2.6)	326	.05	3.4 (3.2)	326	.03
	JSCT	9.5 (1.4)	440	<.001	3363 (1785)	107	<.001	—	—	—	—	—	—

^a^Not applicable.

^b^IGICH: Indira Gandhi Institute of Child Health.

^c^SAM: Project Samraksha.

^d^TSCS: Thalassemia and Sickle Cell Society.

^e^RDT: Rural Development Trust.

^f^JSCT: Jai Shivshakti Center for Thalassemia.

### Other Changes

For each day of work at the thalassemia clinic, a mean 1.3 (range 1.0-1.7) users logged in with at least one record posted from within the center. Multiple log-ins/posts by the same user were counted as 1. The mean records posted per day was 30.6 (range 17.0-53.8).

The total percentage of external consultations ranged from 0% to 92% with a mean of 19.24% (786/4086). Nurses and coordinators from three centers heavily relied on remote advice. Involvement of remote hematologists helped identify 23 patients with SCD and 7 patients with hereditary spherocytosis, all of whom were being inappropriately treated with blood transfusions. This was subsequently corrected.

No nurse initially delegated to work in the thalassemia day care center changed job location during the study period.

Identification of candidates suitable for transplantation led to 391 patients getting human leukocyte antigen-typed and prepared for bone marrow transplantation from these centers. In all, 43 were transplanted with overall survival of 93% and disease-free survival of 77% at a mean cost of US $13,000 per transplant, including pretransplant preparation and late complications [45,46]. For these patients, online software specific for bone marrow transplantation was used [47].

## Discussion

The adoption of an electronic system for patient care was smooth in all centers. The high degree of daily usage (1.3 user log-ins per center and 30.6 posts per day) demonstrates that the ICT platform was well received and operated by the users. We believe that the use of information technology and outcome measurement led to much-needed recognition of the impact created by individuals in the team—especially at a junior level—and may have contributed to the low professional turnover observed in our centers [48].

The ICT platform integrates with the laboratory information systems relieving both health care professionals and parents from the effort of tracking and securing reports. Autogenerated labels for samples, computer-generated blood request forms, and electronic laboratory integration decreased work burden. No additional manpower was recruited just for data management and no additional compensation paid for ICT platform usage. More patients were managed (increase of 0% to 83% with a mean of 18% between centers) with the same staff after the adoption of ICT platform. The cost of using ThalCare was less than US $200 per month per center.

Of all amendments made to chelation doses, 53% were triggered by the ICT platform. Unintended omissions of clinical interventions such as timely dose modification were significantly reduced, even as the clinicians devoted a similar amount of time. We observed better adherence to prescribed regular investigations and better estimation of the blood volume to be transfused.

Ensuring regular supply of blood is a considerable challenge in LMIC and often the family is made responsible to organize blood [49]. The ICT platform helped improve the relationship between treatment center and blood banks as pointed out by the reduced need for third-party blood banks. Regular sharing of data on the turnaround time for blood processing, time between collection and issue of blood products, reporting of transfusion reactions, and careful monitoring of transfusion-transmitted infections allowed the blood banks to implement internal changes meeting the specific requirements of the severe thalassemia clinic.

Often the only medical record available for a child diagnosed with severe thalassemia continues to be notebooks, files, or at best a preprinted diary, which are often soiled, worn out, and prone to being lost. The ICT platform relieves the family from the burden of having to maintain the essential medical history of the child. Unfortunately, so far the uptake of the patient app has been very limited primarily for socioeconomic reasons. This is an area of concern which we continue to work on.

Poverty, ignorance, and illiteracy continue to be significant challenges in LMIC [12,50]. Often the responsibility of adhering to treatment protocol, including getting regular tests done and seeking appointments, is passed on to the family. The socially better-placed families end up getting better quality of care—even as the neglect of the most vulnerable ones continues [51]. Systematic monitoring of each patient’s clinical status allows early identification of those patients who need more attention—decoupling the family’s socioeconomic status from the actual awareness and recognition of the patient’s needs. By achieving this, the ICT platform has the potential to improve equity in access to care.

All blood components were provided free of charge [52] and centers were largely supported by nonprofit organizations mentioned in the Acknowledgment section. The mean overall cost per child was about US $1000 per year. This is a third of the cost of management estimated in India in 2008 (which is likely to have increased with inflation) [53].

With NCD-related health care costs rising and becoming an increasing concern globally, the need for judicious, accountable, and traceable use of resources seems increasingly relevant. The ICT platform enables measurability of the delivery of care, provides a way to maintain an internal check, and to generate outcome benchmarking.

An example of the impact on strategic planning aided by the ICT platform is the decision on purchasing deferoxamine infusion pumps and loaning them to the patients. Similarly, data continues to support the centers in their decision to select the method for leukoreduction of blood products.

The role of ICT in enabling tertiary care in developing countries has been described earlier [47] and challenges on the road to sustainable implementation have been noted [54-56]. Our experience shows successful adoption and sustained use of the ICT platform across five centers in different settings with enhanced outcomes.

Given the general shortage of qualified health care professionals, the ICT platform may have a critical role enabling networking and collaboration. Electronically maintained, well-structured records help overcome wide gaps in the availability of specialists. It is notable that 19% of all consultations were made by health service providers outside the center; the number was as high as 92% for a less experienced center. It is important to highlight that when aided online, the less experienced centers performed as well as their more experienced counterparts. The fact that mismanaged patients were identified and their treatment course corrected by peers from more experienced centers is an example of the possibilities such a model of collaboration brings in. The system can enable that knowledge and best practices are propagated seamlessly allowing centers to be involved in more frequent and well-directed knowledge exchange with peers.

A unique differentiator of this experience has been the fact that unlike the usual practice of limiting the use of ICT to enable collaboration between centers for seeking advice on telemedicine basis or specific periodic interactions, ICT was used for asynchronous teamwork. Apart from being more traceable, this makes it convenient across individual schedules and time zones, thereby creating a sort of patient-specific forum.

In conclusion, our model suggests that computer-assisted treatment planning and performance assessment can significantly improve indexes associated with the effective delivery of care to patients suffering from severe thalassemia. In our experience, a focused context-appropriate, user-driven online IT (Information Technology) tool can have major impact on health care delivery and, importantly, this can occur independently of additional financial and professional resources. Finally, our observation underscores how ICT assists objective outcome reporting, which is the ultimate indicator of quality of care.

## Abbreviations

IGICH: Indira Gandhi Institute of Child Health
JSCT: Jai Shivshakti Center for Thalassemia
ICT: information and communication technology
LMIC: low- and middle-income countries
NCD: noncommunicable diseases
RDT: Rural Development Trust
SAM: Project Samraksha
SCD: sickle cell disease
TSCS: Thalassemia and Sickle Cell Society

## References

[1] Poushter J (2016). Pew Research Center.

[2] Park H (2016). Health informatics in developing countries: a review of unintended consequences of IT implementations, as they affect patient safety and recommendations on how to address them. Yearb Med Inform.

[3] Chavannes NH, Du Puy RS, Bai C (2015). Suggestions for health information technology trials for respiratory disorders in low- and middle-income country settings: what can we learn from trials in high-income country settings?. NPJ Prim Care Respir Med.

[4] Kahouei M, Zadeh JM, Roghani PS (2015). The evaluation of the compatibility of electronic patient record (EPR) system with nurses' management needs in a developing country. Int J Med Inform.

[5] Agarwal S, Perry HB, Long L, Labrique AB (2015). Evidence on feasibility and effective use of mHealth strategies by frontline health workers in developing countries: systematic review. Trop Med Int Health.

[6] Huckvale C, Car J, Akiyama M, Jaafar S, Khoja T, Bin KA, Sheikh A, Majeed A (2010). Information technology for patient safety. Qual Saf Health Care.

[7] Heeks R (2002). Information systems and developing countries: failure, success, and local improvisations. Inf Soc.

[8] Moucheraud C, Schwitters A, Boudreaux C, Giles D, Kilmarx PH, Ntolo N, Bangani Z, St Louis ME, Bossert TJ (2017). Sustainability of health information systems: a three-country qualitative study in southern Africa. BMC Health Serv Res.

[9] Irinoye OO, Ayandiran EO, Fakunle I, Mtshali N (2013). Nurses' perception and barriers to use of information communication technology in a teaching hospital in Nigeria. Comput Inform Nurs.

[10] (2015). World Health Statistics 2015.

[11] Overdorf J (2012). Public Radio International.

[12] Balarajan Y, Selvaraj S, Subramanian S (2011). Health care and equity in India. Lancet.

[13] Iskrov G, Stefanov R (2016). Ensuring transparency and consistency in the value assessment of cancer therapies. J Clin Oncol.

[14] Elzawawy AM (2015). Could African and low- and middle-income countries contribute scientifically to global cancer care?. J Glob Oncol.

[15] The Lancet (2017). Health in India, 2017. Lancet.

[16] de Silva S, Fisher CA, Premawardhena A, Lamabadusuriya SP, Peto TE, Perera G, Old JM, Clegg JB, Olivieri NF, Weatherall DJ (2000). Thalassaemia in Sri Lanka: implications for the future health burden of Asian populations. Sri Lanka Thalassaemia Study Group. Lancet.

[17] Riewpaiboon A, Nuchprayoon I, Torcharus K, Indaratna K, Thavorncharoensap M, Ubol B (2010). Economic burden of beta-thalassemia/Hb E and beta-thalassemia major in Thai children. BMC Res Notes.

[18] Scalone L, Mantovani LG, Krol M, Rofail D, Ravera S, Bisconte MG, Borgna-Pignatti C, Borsellino Z, Cianciulli P, Gallisai D, Prossomariti L, Stefàno I, Cappellini MD (2008). Costs, quality of life, treatment satisfaction and compliance in patients with beta-thalassemia major undergoing iron chelation therapy: the ITHACA study. Curr Med Res Opin.

[19] Payne KA, Rofail D, Baladi J, Viala M, Abetz L, Desrosiers M, Lordan N, Ishak K, Proskorovsky I (2008). Iron chelation therapy: clinical effectiveness, economic burden and quality of life in patients with iron overload. Adv Ther.

[20] Weidlich D, Kefalas P, Guest JF (2016). Healthcare costs and outcomes of managing β-thalassemia major over 50 years in the United Kingdom. Transfusion.

[21] Ho W, Lin K, Wang J, Hwang J, Chung C, Lin D, Jou S, Lu M, Chern JP (2006). Financial burden of national health insurance for treating patients with transfusion-dependent thalassemia in Taiwan. Bone Marrow Transplant.

[22] Weatherall DJ (2010). The inherited diseases of hemoglobin are an emerging global health burden. Blood.

[23] Mohanty D, Colah RB, Gorakshakar AC, Patel RZ, Master DC, Mahanta J, Sharma SK, Chaudhari U, Ghosh M, Das S, Britt RP, Singh S, Ross C, Jagannathan L, Kaul R, Shukla DK, Muthuswamy V (2013). Prevalence of β-thalassemia and other haemoglobinopathies in six cities in India: a multicentre study. J Community Genet.

[24] Agarwal MB (2009). Advances in management of thalassemia. Indian J Pediatr.

[25] Weatherall DJ (2011). The challenge of haemoglobinopathies in resource-poor countries. Br J Haematol.

[26] Galanello R, Origa R (2010). Beta-thalassemia. Orphanet J Rare Dis.

[27] Rund D (2016). Thalassemia 2016: modern medicine battles an ancient disease. Am J Hematol.

[28] Modell B, Darlison M (2008). Global epidemiology of haemoglobin disorders and derived service indicators. Bull World Health Organ.

[29] Prakash A, Aggarwal R (2012). Thalassemia major in adults: short stature, hyperpigmentation, inadequate chelation, and transfusion-transmitted infections are key features. N Am J Med Sci.

[30] Verma IC, Saxena R, Kohli S (2011). Past, present & future scenario of thalassaemic care & control in India. Indian J Med Res.

[31] Cappellini M, Cohen A, Porter J, Taher A, Viprakasit V (2014). Guidelines for the management of transfusion dependent thalassaemia (TDT).

[32] Yin X, Wu Z, He Y, Zhou T, Zhou Y, Zhang XH (2011). Treatment and complications of thalassemia major in Guangxi, Southern China. Pediatr Blood Cancer.

[33] Hashemizadeh H, Noori R, Kolagari S (2012). Assessment hepatomegaly and liver enzymes in 100 Patients with beta thalassemia major in Mashhad, Iran. Iran J Ped Hematol Oncol.

[34] Olivieri NF, Brittenham GM (2013). Management of the thalassemias. Cold Spring Harb Perspect Med.

[35] Lucarelli G, Galimberti M, Polchi P, Angelucci E, Baronciani D, Giardini C, Politi P, Durazzi SM, Muretto P, Albertini F (1990). Bone marrow transplantation in patients with thalassemia. N Engl J Med.

[36] Mathews V, George B, Deotare U, Lakshmi K, Viswabandya A, Daniel D, Chandy M, Srivastava A (2007). A new stratification strategy that identifies a subset of class III patients with an adverse prognosis among children with beta thalassemia major undergoing a matched related allogeneic stem cell transplantation. Biol Blood Marrow Transplant.

[37] Thacker N (2007). Prevention of thalassemia in India. Indian Pediatr.

[38] Ancker JS, Edwards A, Nosal S, Hauser D, Mauer E, Kaushal R, with the HITEC Investigators (2017). Effects of workload, work complexity, and repeated alerts on alert fatigue in a clinical decision support system. BMC Med Inform Decis Mak.

[39] Aydinok Y, Kattamis A, Viprakasit V (2014). Current approach to iron chelation in children. Br J Haematol.

[40] Taher A, El-Beshlawy A, Elalfy MS, Al Zir K, Daar S, Habr D, Kriemler-Krahn U, Hmissi A, Al Jefri A (2009). Efficacy and safety of deferasirox, an oral iron chelator, in heavily iron-overloaded patients with beta-thalassaemia: the ESCALATOR study. Eur J Haematol.

[41] Mishra AK, Tiwari A (2013). Iron overload in beta thalassaemia major and intermedia patients. Maedica (Buchar).

[42] World Health Organization.

[43] World Health Organization.

[44] Dhingra B, Sharma S, Mishra D, Kumari R, Pandey RM, Aggarwal S (2010). Normal values of liver and spleen size by ultrasonography in Indian children. Indian Pediatr.

[45] Faulkner L, Uderzo C, Khalid S, Marwah P, Soni R, Yaqub N, Amanat S, Fatima I, Gilani SK, Zahra T, Ramprakash S, Gooneratne L, Dissanayake R, Williams S, Rathnayake W, Srinivas R, Sedai A, Kumari A, Parmar L, Dhanya R, Agarwal RK (2017). ATG vs thiotepa with busulfan and cyclophosphamide in matched-related bone marrow transplantation for thalassemia. Blood Adv.

[46] Agarwal R, Kumari A, Sedai A, Parmar L, Dhanya R, Faulkner L (2017). The case for high resolution extended 6-loci HLA typing for identifying related donors in the Indian subcontinent. Biol Blood Marrow Transplant.

[47] Agarwal RK, Sedai A, Dhimal S, Ankita K, Clemente L, Siddique S, Yaqub N, Khalid S, Itrat F, Khan A, Gilani SK, Marwah P, Soni R, Missiry ME, Hussain MH, Uderzo C, Faulkner L (2014). A prospective international cooperative information technology platform built using open-source tools for improving the access to and safety of bone marrow transplantation in low- and middle-income countries. J Am Med Inform Assoc.

[48] Bhattacharya I, Ramachandran A (2015). A path analysis study of retention of healthcare professionals in urban India using health information technology. Hum Resour Health.

[49] Anand A (2015). BBC News.

[50] Rajan K, Kennedy J, King L (2013). Is wealthier always healthier in poor countries? The health implications of income, inequality, poverty, and literacy in India. Soc Sci Med.

[51] Gwatkin DR, Bhuiya A, Victora CG (2004). Making health systems more equitable. Lancet.

[52] (2014). Guidelines for Recovery of Processing Charges for Blood and Blood Components.

[53] Ghosh K, Colah R (2008). Control and Management of Thalassemia and Other Hemoglobinopathies in the Indian Subcontinent : Synoptic Views.

[54] Luna D, Almerares A, Mayan JC, González BD, Otero C (2014). Health informatics in developing countries: going beyond pilot practices to sustainable implementations: a review of the current challenges. Healthc Inform Res.

[55] Lewis T, Synowiec C, Lagomarsino G, Schweitzer J (2012). E-health in low- and middle-income countries: findings from the Center for Health Market Innovations. Bull World Health Organ.

[56] Kostkova P (2015). Grand challenges in digital health. Front Public Health.

